# Theoretical Analysis of Polynuclear Zinc Complexes Isolobally Related to Hydrocarbons

**DOI:** 10.3390/ijms232314858

**Published:** 2022-11-28

**Authors:** Regla Ayala, Agustín Galindo

**Affiliations:** 1 Departamento de Química Inorgánica, Facultad de Química, Universidad de Sevilla, Aptdo 1203, 41071 Sevilla, Spain; 2 Instituto de Ciencia de Materiales de Sevilla-CSIC, Avda. Américo Vespucio 49, 41092 Sevilla, Spain

**Keywords:** zinc, DFT, QTAIM, dizinc, organometallic complexes

## Abstract

Based on the isolobal analogy of ZnCp (Cp = η^5^-C_5_H_5_) and ZnR (R = alkyl or aryl group) fragments with hydrogen atom and fragment [Zn(CO)_2_] with a CH_2_ carbene, the following complexes [(ZnCp)_2_{µ-Zn(CO)_2_}], **1**, [(ZnPh)_2_{µ-Zn(CO)_2_}], **2**, [(ZnPh){µ-Zn(CO)_2_}(ZnCp)], **3**, [(ZnCp)_2_{µ-Zn_2_(CO)_4_}], **4**, [(ZnPh)_2_{µ-Zn_2_(CO)_4_}], **5**, [(ZnPh){µ-Zn(CO)_2_}_2_(ZnCp)], **6**, [Zn_3_(CO)_6_], **7** and [Zn_5_(CO)_10_], **8**, were built. These polynuclear zinc compounds are isolobally related to simple hydrocarbons (methane, ethane, cyclopropane and cyclopentane). They have been studied by density functional theory (DFT) and quantum theory of atoms in molecules (QTAIM) to compare the nature and topology of the Zn–Zn bond with previous studies. There are bond critical points (BCPs) between each pair of adjacent Zn centers in complexes **1**–**8** with Zn–Zn distances within the range 2.37–2.50 Å. The nature of the Zn–Zn bond in these complexes can be described as polar rather than pure covalent bonds. Although in a subtle way, the presence of different ligands and zinc oxidation states introduces asymmetry and polarity in the Zn–Zn bond. In addition, the Zn–Zn bond is delocalized in nature in complex **7** whereas it can be described as a localized bond for the remaining zinc complexes here studied.

## 1. Introduction

The characterization by Carmona and co-workers of complex [Zn_2_Cp*_2_] (Cp* = η^5^-C_5_Me_5_), a species showing a dizinc unit directly bonded with a formal oxidation state of I, opened a new chapter in the chemistry of the zinc element [1,2]. After this report, the until then unknown Zn–Zn bond immediately attracted a great interest and Carmona’s research group [3,4,5] and some others [6,7,8,9,10,11,12,13,14] rapidly expanded the number of well-characterized complexes containing this bond [15,16]. This experimental development of dizinc chemistry was almost simultaneously accompanied by a good number of theoretical studies devoted to exploring the main features of this new bond (see, for example, reference [17]). The theoretical interest in the dizinc bond was then extended to other examples different from those with the Zn(I)–Zn(I) unit. Thus, single and multiple bonds have been proposed and theoretically investigated between Zn^0^ atoms [18,19,20,21,22,23]. 

Following our interest in the theoretical analysis of Zn–Zn bonded molecules [24,25,26], we decide to explore the nature of this bond in polynuclear zinc complexes that are isolobally related to simple hydrocarbons. For this purpose, we used the isolobal analogy of ZnCp and ZnR fragments with the H atom and that of the fragment [Zn(CO)_2_] with a CH_2_ carbene. Here, we report density functional theory (DFT) and quantum theory of atoms in molecules (QTAIM [27,28,29,30,31]) studies of several polynuclear zinc models isolobally related to methane ([(ZnCp)_2_{µ-Zn(CO)_2_}], **1**, [(ZnPh)_2_{µ-Zn(CO)_2_}], **2**, and [(ZnPh){µ-Zn(CO)_2_}(ZnCp)], **3**), ethane ([(ZnCp)_2_{µ-Zn_2_(CO)_4_}], **4**, [(ZnPh)_2_{µ-Zn_2_(CO)_4_}], **5**, and [(ZnPh){µ-Zn(CO)_2_}_2_(ZnCp)], **6**), cyclopropane ([Zn_3_(CO)_6_], **7**) and cyclopentane ([Zn_5_(CO)_10_], **8**). This analogy implies the combination of different oxidation states and ligands what can introduce asymmetry within the Zn–Zn bond in these complexes. The former can have significant implications for their reactivity and applications. The specific nature of the Zn–Zn bonds in these complexes will be discussed and compared with those from related studies. 

## 2. Results and Discussion

### 2.1. The Zn–Zn Bond: Experimental Data

X-ray crystallography is the experimental technique widely used for the authentication of a zinc–zinc bond. A search in the Cambridge Structural Database (CSD) [32] of complexes containing Zn–Zn bonds without bridging ligands yields approximately 60 hits, in which the distances of the Zn–Zn bonds cover the range 2.29–2.45 Å (Figure S1 shows the corresponding histogram in the Supplementary Material). The mean value found for the Zn–Zn bond is 2.35 Å, which is indicative of a covalent bond taking into account the covalent radius of the single-bonded zinc (1.22 Å [33]). Distances longer than 2.45 Å have been described in complexes in which the dizinc bond acts as a ligand [24,34,35], and in several polynuclear clusters [36,37,38,39]. In any case, the value of 2.68 Å (double of the metallic zinc radius, 1.34 Å) can be considered as the limit to the existence of a Zn–Zn interaction. In addition to experimental data, theoretical methods will certainly aid in defining the nature of a Zn–Zn bond, in particular for those examples showing weak interactions. For example, we proposed a range of 2.44–2.58 Å for elongated dizinc bonds [24]. 

### 2.2. The Isolobal Analogies of CpZn, PhZn and Zn(CO)_2_ Fragments

**Scheme 1:**
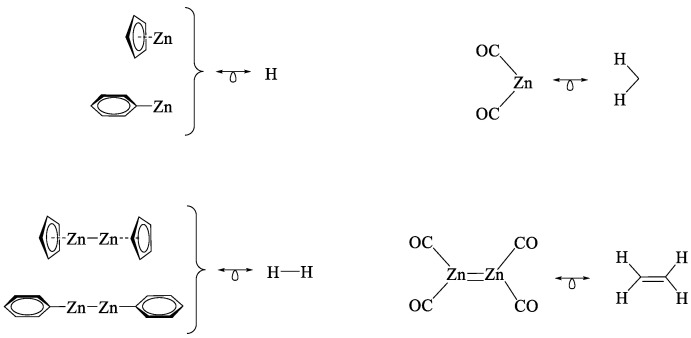
Isolobal relationships of CpZn, PhZn and Zn(CO)_2_ fragments.

The isolobal relationship between ZnCp and ZnR with the H atom was first recognized by Fischer, Frenking and co-workers [40,41,42]. The frontier MOs of these *d*^10^*s*^1^-fragments are similar, the SOMO being constituted by a *σ* hybrid with an important *s* contribution. Essentially, the dizinc bond in the dimers can be qualitatively interpreted as a *s*-*s* overlap. In fact, Carmona [1,2] or Power [6] complexes are isolobally related to the dihydrogen molecule (Scheme 1). We previously used this relationship to investigate the behavior of compounds [Zn_2_Cp_2_] and [Zn_2_Ph_2_] as coordinated ligands in various ML_n_ transition metal fragments [24]. Alternatively, Alvarez and coworkers first analyzed the double bond between two zinc(0) atoms in several dizinc complexes of [Zn_2_(L)_4_] type (L = neutral ligand) [19]. The ZnL_2_ monomers displayed non-linear geometries, like a carbene, and were found as energy minima in their triplet states. Furthermore, the ZnL_2_ and CH_2_ groups show similar shapes of their singly occupied MOs. Consequently, the compound [Zn_2_(CO)_4_] is isolobally related to ethylene because the Zn(CO)_2_ fragment is isolobally related to a CH_2_ carbene (Scheme 1) [19]. Based on such a relationship, we previously studied the behavior of the [Zn_2_(CO)_4_] species as a ligand versus different ML_n_ transition metal fragments [25]. 

### 2.3. The Zn–Zn Bond in Complexes ***1***–***6***

**Scheme 2:**
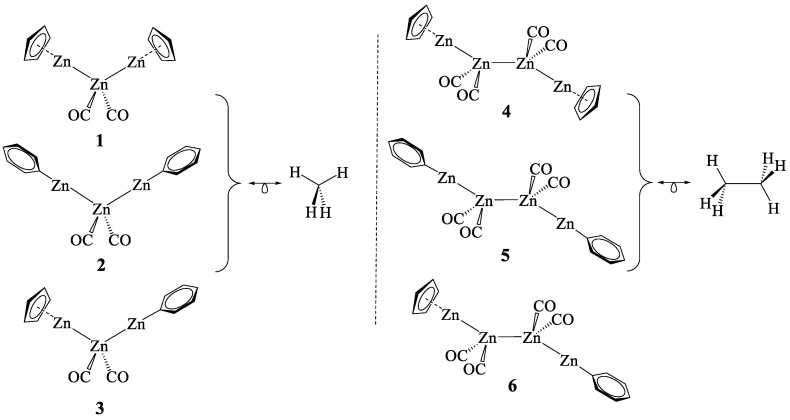
Polynuclear zinc complexes isolobally related to methane and ethane.

**Figure 1:**
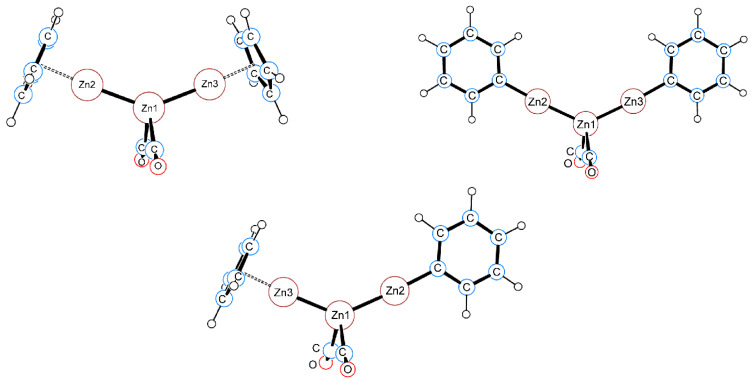
Optimized structures of complexes **1**–**3**, isolobally related to methane.

**Figure 2:**
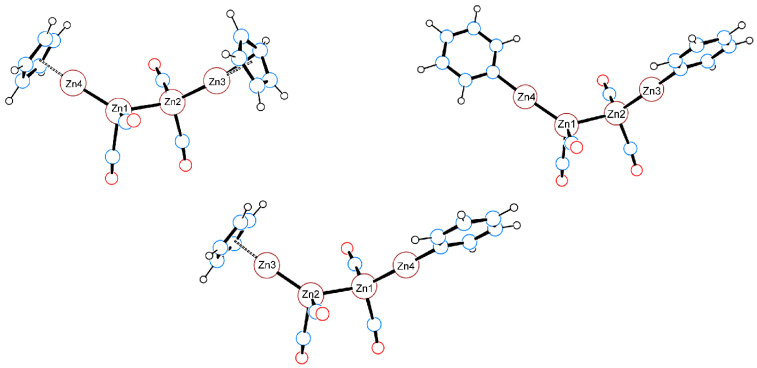
Optimized structures of complexes **4**–**6**, isolobally related to ethane.

The hydrogen atom and the simplest CH_2_ carbene are the indispensable pieces of the puzzle to build up hydrocarbon molecules. With this idea in mind, we decided to assemble several zinc fragments to obtain polynuclear zinc complexes that were isolobally related to methane and ethane, as representative examples of simple hydrocarbons (Scheme 2). Complexes **1**–**6** were optimized without symmetry constraints. The resulting structures are shown in Figure 1 and Figure 2, while selected structural parameters are collected in Table 1. 

Complexes **1**–**3**, isolobally related to methane, are characterized by a bent trizinc Zn–Zn–Zn unit with angles within the 129–139° range (Table 1). Particularly relevant to complexes **1**–**3**, which show 0 and I as formal zinc oxidation states, is the report of a complex with a linear L-Zn–Zn–Zn–L moiety (where L is a bulky amide) in which the central naked Zn(0) atom bonds to its adjacent Zn(I) neighbors. This complex shows a Zn–Zn distance of 2.3840(12) Å [43]. The Zn–Zn lengths calculated in **1**–**3** (around 2.40 Å (Table 1)) compare well with this experimental value and are indicative of a single dizinc bond. Additionally, Zn-C_Ph_ and Zn-Cp_centroid_ lengths (*ca*. 1.97 and 1.96 Å) are well correlated with the analogous distances reported for the complexes [Zn_2_Ar’_2_] and [Zn_2_Cp_2_*]. As expected, the Zn–Zn–C_Ph_ and Zn–Zn–Cp_centroid_ angles are close to 180°. The presence of adjacent zinc ions to the Zn(CO)_2_ unit in **1**–**3** stabilizes the coordination of CO ligands to the central zinc atom. This is confirmed by the optimization of hypothetical complexes [ZnR_2_(CO)_2_] (R = H, Me), which are isolobally related to **1**–**3**, because the optimized result of this optimization is the linear species [ZnR_2_] and two non-coordinated CO molecules. 

Regarding the metal–metal bond, the HOMO of **1** is quite similar to that described for the complex L–Zn–Zn–Zn–L [43]. This orbital is mainly constituted by the overlap of a *sp* orbital of the central zinc center with the *s* orbitals of the terminal zinc atoms (Figure S2). In fact, the NBO analysis of the Zn–Zn bonds of **1** reveals a major contribution of the *s* orbital (84%) of terminal Zn atoms and the mixture of *s* and *p* orbitals for the central Zn atom (37% and 63%, respectively). When the terminal Zn atom is bonded to the phenyl substituent, an increase of the participation of the *p* orbital is observed in the HOMOs of **2** and **3** (Figure S2). The HOMOs of complexes **1**–**3** and other MOs involved in the Zn–Zn interactions are collected in Figure S2. 

Complexes **4**–**6**, isolobally related to ethane, are characterized by a non-linear tetrazinc Zn–Zn–Zn–Zn unit with angles within the 129–135° range (Table 1) and torsion angles between 81° and 94°. The Zn^I^–Zn^0^ bond distances are comparable to those calculated for **1**–**3** and shorter than those of the central Zn^0^–Zn^0^ bonds (Table 1), the latter fall in the range of the elongated dizinc bonds. In fact, in all complexes, the Cp(Ph)Zn–Zn(CO)_2_ distances are shorter than the (CO)_2_Zn–Zn(CO)_2_ ones by several tenths of angstroms. In turn, Zn–Zn distances involving Ph ligands are longer than those with Cp ligands as occurs in the Zn_2_Cp_2_ and Zn_2_Ph_2_ compounds [24]. The MOs involved in all Zn–Zn interactions are shown in Figure S3. For example, in **4** there are three MOs (HOMO, HOMO-5 and HOMO-6) in which the terminal Zn atoms mainly contribute with their *s* orbitals, while for the central Zn atoms the major contribution comes from *p* orbitals (Figure S3). The exception is the delocalized HOMO-6 in which the *s* orbital is the key contribution for all Zn atoms. 

### 2.4. QTAIM Analysis of the Zn–Zn Bond in Complexes ***1***–***6***

The application of Bader’s Quantum Theory of Atoms in Molecules allows the study of important information about different chemical properties in a complex [27,28,29,30,31]. Selected local and integral topological properties of the electron density for complexes **1**–**6** are presented in Table 2. Topological graphs showing bond paths (BPs) and critical points (CPs) are collected in Figure S4 (Supplementary Material). The QTAIM study showed that there is a bond critical point (BCP) between each pair of adjacent Zn centers, which can be interpreted as a Zn–Zn bond. BPs are straight lines in all cases, no curvatures have been found. The BCPs are located at the midpoint of the Zn–Zn distance when the same ligands are bonded to the metal centers, that is, in the case of (CO)_2_Zn–Zn(CO)_2_ moieties. This can be observed even in complex **6** which accounts for different terminal fragments. On the contrary, the BCP of the Ph(Cp)Zn–Zn(CO)_2_ is closer to Ph(Cp)Zn than to the Zn(CO)_2_ unit. This behavior has already been observed in mixed complexes [LZn_2_L’] involving Zn–Zn bonds [26]. 

The electron density (ρ) and its laplacian (▽^2^ρ) at the BCP are the parameters normally used to determine the nature and extent of the bonding between two atoms. The well-known trends associated with these magnitudes cannot be applied in the case of polymetallic transition complexes due to the diffuse electron distributions of metal–metal bonds [44]. As expected for heavy metal atoms, ρ_BCP_ and ▽^2^ρ_BCP_ at Zn–Zn bonds give rise to low values that rule out their use for a bonding classification. In this context, the energetic parameters per electron are more meaningful, such as the kinetic energy density G(r)/ρ_BCP_ and the total energy density H(r)/ρ_BCP_ [44,45,46,47,48]. The positive values, smaller than unity, obtained for G(r)/ρ_BCP_ ratios imply the presence of open-shell Zn–Zn interactions in all the complexes. This is also supported by the values of the |V_BCP_|/G_BCP_ ratio between 1 and 2 corresponding to a transient region related with polar covalent Zn–Zn bonds. The bonding degree (BD), H_BCP_/ρ_BCP_, indicates the degree of covalency, in such a way that the greater its magnitude, the more covalent and stronger the bond is. The negative, albeit small, values of the BD parameter can be taken as indicative of the covalency of the bond, although the degree must be moderate. 

A first inspection of Table 2 indicates that the Zn–Zn bonds in complexes **1**–**6** are rather similar to each other. Nevertheless, a deep analysis allows one to find differences that are determined by the influence of the ligands and the formal oxidation states of the Zn centers. ρ_BCP_ and ▽^2^ρ_BCP_ at Zn–Zn BCPs are larger for fragments involving the Cp ligands than for the Ph ligands. The smallest value is obtained for the (CO)_2_Zn–Zn(CO)_2_ bonds. Clearly, the presence of a double bond between the central Zn atoms observed in previous complexes [19,25] does not appear in complexes **4**–**6**, isolobally related to ethane. This trend runs in parallel with the Zn–Zn distances previously commented. In addition, the ellipticity at the BCP, ε_BCP_, measures the extent to which the electron density is preferentially accumulated in a given plane containing the bond path. The ε_BCP_ at the Zn–Zn BCP decreases when going from PhZn–Zn(CO)_2_ > CpZn–Zn(CO)_2_ > (CO)_2_Zn–Zn(CO)_2_. These values are lower than those found in other theoretical Zn–Zn compounds involving the same fragments but a certain double bond character [24]. 

The δ(Zn,Zn) delocalization index alludes to the number of electron pairs shared between the Zn atoms. It is typically related to the bonding mechanism and only indirectly to the interaction strength, which is revealed by the value of the electronic density integrated over the whole interatomic surface ∫_Zn∩Zn_ρ. δ(Zn,Zn) values are in the range 0.496–0.781, suggesting again the presence of polar rather than pure covalent bonds. This agrees with the Wiberg bond indexes obtained in the NBO analyses. WBI indicates that the introduction of a Zn(CO)_2_ fragment between either the CpZn–Zn(CO)_2_ or PhZn–Zn(CO)_2_ units weakens the Zn–Zn bond. 

### 2.5. The Zn–Zn Bond in Complexes ***7*** and ***8***

**Figure 3:**
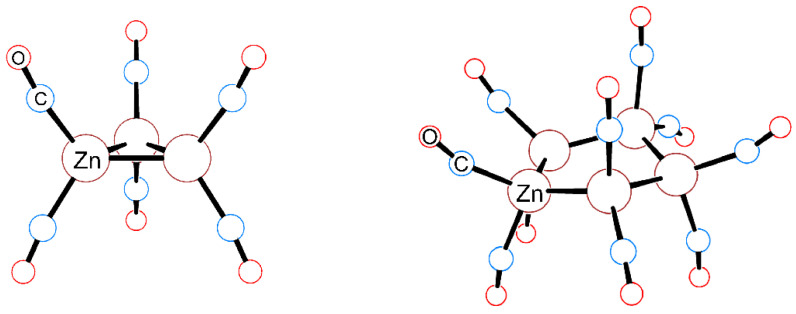
Optimized structures of complexes **7** and **8**, isolobally related to cyclopropane and cyclopentane, respectively.

Using the isolobal relationship between [Zn(CO)_2_] and CH_2_, we have also studied a couple of examples of compounds related to simple cyclic hydrocarbons, such as cyclopropane and cyclopentane. Complexes [Zn_3_(CO)_6_], **7** and [Zn_5_(CO)_10_], **8**, were optimized without symmetry constraints. The resulting structures are shown in Figure 3, while the selected structural parameters are collected in Table 3. The number of well characterized molecular Zn(0) complexes is small [49,50] and there are no experimental examples of covalent Zn(0)–Zn(0) bonds. Precedents related with these compounds are the calculated species [Zn_2_(CO)_4_] (Zn = Zn bond of 2.273 Å [19]) and also Zn−Zn(CO) and Zn−Zn(CO)_2_ compounds, with Zn−Zn distances within the range of 2.42–2.54 Å depending on the computational method applied [18]. The Zn–Zn bond lengths calculated in these cyclic models are lower than 2.50 Å, specifically between the upper part of the range of single covalent bonds and the lower range of elongated dizinc bonds. In any case, they are significantly lower than the value found for diatomic van der Waals Zn_2_ species (4.19 Å), which was characterized in the gas phase by UV spectroscopy [51]. MOs of complexes **7** and **8** involved in Zn–Zn interactions are collected in Figure S5.

### 2.6. QTAIM Analysis of the Zn–Zn Bond in Complexes ***7***–***8***

Selected local and integral topological properties of the electron density for complexes **7**–**8** are presented in Table 4. For the sake of comparison, [Zn_2_(CO)_4_] results have also been included. Topological graphs showing bond paths (BPs) and critical points (CPs) are collected in Figure S4 (Supplementary Material). Zn–Zn BCPs with linear BPs are found between each pair of adjacent Zn centers. The topological and integral parameters of complexes **7** and **8** indicate that the Zn–Zn bond can be described as a polar covalent bond. In addition, there is a ring critical point (RCP) in the center of the cycle in both complexes. It should be mentioned that ε_BCP_ at Zn–Zn BCP is very high and could be interpreted as a certain double bond character. Nevertheless, it must be considered as an important delocalization of the Zn–Zn bond among the three zinc atoms in complex **7**. The Source Function described in the next section reinforces this idea. 

A comparison with the non-cyclic [Zn_2_(CO)_4_] complex [19,25] in which a double Zn–Zn bond was identified can be very instructive. Complexes **7** and **8** present longer distances, lower δ(Zn,Zn) and BD parameters what reflects a weakening of the Zn–Zn bond, and allow us to estimate the lack of a double bond in these complexes. The comparison of Table 2 and Table 3 reveals that the (CO)_2_Zn–Zn(CO)_2_ bonds present similar stabilities in the non-cyclic and cyclic complexes and they are weaker in both cases than the PhZn–Zn(CO)_2_ or CpZn–Zn(CO)_2_ interactions. 

### 2.7. The Source Function and Electron Localization Function in Complexes ***1***–***8***

The Source Function (SF) using the Zn–Zn BCP as reference gives information about the contribution of each atom in the complex to the Zn–Zn bond. Thus, the SF at the Zn–Zn BCP can supply information about asymmetry and polarity of the bond. Table 5 collects the SF at Zn–Zn BCPs for complexes **1**–**8**. The contribution of the Zn bonded atoms is different depending on the nature of the ligands. When two different fragments are bonded, the Zn atom that interacts with the Cp or Ph ligands contributes largely to the bond compared to Zn that interacts with CO ligands. Nonetheless, the contribution of both Zn centers in the case of the (CO)_2_Zn–Zn(CO)_2_ bond is the same. The participation is rather independent of the isolobal analogy, that is, the inclusion of an additional (CO)_2_Zn–Zn(CO)_2_ unit does not affect the SF. It is also similar between linear **4**–**6** and cyclic **7**–**8** compounds. The different contributions agree with the position of the BCP in Zn–Zn BP. In complexes **1**–**6**, the participation of the Zn that does not take part of the reference BCP is quite low. In contrast, the SF for the O atoms of CO ligands that interact with the bonded Zn is significant (~7%) being a bit larger in the case of (CO)_2_Zn–Zn(CO)_2_ bonds. Nevertheless, it is less important than that found in L_n_MZn_2_(CO)_4_ where certain double character is present (7% vs. 20%) [25].

In the case of the cyclic complexes, both present a RCP in which all Zn atoms participate in a symmetrical way. However, the contribution of the Zn atoms non-bonded in the BCP taken as reference depends on the complex. On one hand, the contribution of this Zn in the complex **7** is significant (7.86%). On the other hand, it is rather low for complex **8** (<1%). In fact, the contribution has a negative sign what implies that these Zn atoms subtract density from the Zn–Zn bond. The main difference between both cycles is the lack of planarity in complex **8**. 

It is worth studying if there is a π back donation from the Zn metal to the CO ligand. The detection of backdonation is not straightforward, but the analysis of delocalization index δ(Zn,O) may shed light on this respect. Previous results of δ(Zn,O) where π back donation is present are in the order of 0.2 [24,52], however, this parameter decreases down to 0.05 in the absence of backdonation [47,52]. Our complexes present δ(Zn,O) around 0.07 what can be interpreted as a negligible π back donation. This is confirmed by the calculated ν(CO) frequencies of **1**–**8** that are close to the value of free CO (see Table S1 at Supplementary Material). 

The Electron Localization Function (ELF) gives information about the region of the space where the electrons are localized [53,54]. ELF basins are related to electronic domains and give rise to the idea of Lewis structures and valence shell electronic pair repulsion geometries. Two types of basins can be found: core basins around the nuclei and valence basins in the remaining space. Core basins correspond to the inner atomic shell density, whereas the valence basin density is organized around and between the core basins. The valence basins are characterized by a synaptic order, that is, the number of core basins with which it has a boundary. Monosynaptic basins are the signature of lone pair electrons, whereas polysynaptic basins represent bi-, tri- or, in general, polycentric bonds. Valence basins with a synaptic order larger than 1 imply shared electrons. The population of electrons in a basin can be obtained by the integration of the electron density of the basin. 

The two-dimensional ELF contours and the three-dimensional isosurfaces for complexes **1**–**8** are displayed in Figures S6 and S7, respectively (Supplementary Material). Table 6 collects the polysynaptic basins among Zn centers along with ELF basin population in complexes **1**–**8**. It can be seen that all of the complexes present disynaptic Zn–Zn basins, V(Zn,Zn), between adjacent Zn atoms, in agreement with previous results. The electron population follows the trend V(Zn_Cp_-Zn_CO_) > V(Zn_Ph_-Zn_CO_) > V(Zn_CO_-Zn_CO_) consistent with the degree of interaction between metal centers. This conclusion is the same as that drawn from the previous QTAIM and NBO analyses. Additionally, there is a trisynaptic basin with a population of 0.95 e^−^ in complex **7** that indicates delocalization between the three Zn centers. This fact was also reflected in the ε_BCP_ and SF of this compound previously commented. On the contrary, the absence of tri- or higher synaptic metal order in complex **8** again reinforces the idea of localization in this case. As mentioned before, this can be due to the lack of planarity in this complex. For the sake of comparison, the results of [Zn_2_(CO)_4_] have also been included in Table 6. The decrease in the average electron population in complexes **1**–**8** with respect to the [Zn_2_(CO)_4_] complex clearly confirms the presence of a single bond in the isolobal analogies studied here rather than a certain character of the double bond. 

## 3. Materials and Methods

Quantum chemical optimizations on the basis of the density functional theory (DFT) at the TPSSh/Def2TZVPP level of theory were carried out [55,56]. This level of computation has already been used in the description of similar complexes [24]. Additionally, the comparison with MP2 results (see Table S2 in Supplementary Material) was also carried out to ensure the suitability of our methodology. The optimized geometries of all the compounds were characterized as energy minima by non-existent imaginary frequencies. Electronic calculations were performed using Gaussian16 rev. C01 program [57]. The bonding characteristics were analyzed using the natural bond orbital (NBO) theory. This was achieved with the NBO package 3.1 included in the Gaussian16 suite of programs [58]. Topology parameters were studied within the framework of the QTAIM method implemented in AIMALL program [59] and the Multiwfn program [60]. Coordinates of all optimized compounds are collected in the Supplementary Material (Table S3).

## 4. Conclusions

A DFT and QTAIM study of the dizinc bond in several Zn-complexes has been carried out. These are [(ZnCp)_2_{µ-Zn(CO)_2_}], **1**, [(ZnPh)_2_{µ-Zn(CO)_2_}], **2**, [(ZnPh){µ-Zn(CO)_2_}(ZnCp)], **3**, [(ZnCp)_2_{µ-Zn_2_(CO)_4_}], **4**, [(ZnPh)_2_{µ-Zn_2_(CO)_4_}], **5**, [(ZnPh){µ-Zn(CO)_2_}_2_(ZnCp)], **6**, [Zn_3_(CO)_6_], **7** and [Zn_5_(CO)_10_], **8**, which were built using the isolobal analogy of ZnCp (Cp = η^5^-C_5_H_5_) and ZnPh fragments with H atom and fragment [Zn(CO)_2_] with CH_2_. Thus, **1**–**3** are isolobally related to methane, **4**–**6** to ethane and **7**–**8** to cyclopropane and cyclopentane, respectively. The calculated Zn–Zn distances are typical of single bonds (2.37–2.50 Å range), and this was confirmed by the presence of BCPs between each pair of adjacent Zn atoms. The shortest is found in Zn^I^-Zn^0^ bonds when a CpZn fragment is present (CpZn–Zn(CO)_2_), while longer distances are found for Zn^0^-Zn^0^ bonds ((CO)_2_Zn–Zn(CO)_2_). This sequence of calculated distances is similar to the order found for ρ_BCP_ and ▽^2^ρ_BCP_ parameters at Zn–Zn BCPs. This also agrees with the Wiberg bond indexes, which show that the introduction of a Zn(CO)_2_ fragment between the CpZn–Zn(CO)_2_ or PhZn–Zn(CO)_2_ units weakens the Zn–Zn bond. The nature of Zn–Zn bond according to δ(Zn,Zn) values (range 0.496–0.781) suggests the presence of polar rather than pure covalent bonds. Although in a subtle way, the presence of different ligands and zinc oxidation states introduces asymmetry and polarity in the Zn–Zn bond. SF and ELF analysis reveal that the Zn–Zn bond is delocalized in nature in complex 7 whereas it can be described as a localized bond for the remaining zinc complexes isolobally related to hydrocarbons here studied.

## Figures and Tables

**Table 1:** Selected structural parameters (bond distances, Å, and angles, °) of complexes **1**–**6**.

	1	2	3	4	5	6
Zn–Zn	2.386	2.416	2.392, 2.413 ^a^	2.375, 2.491 ^a^	2.403, 2.498 ^a^	2.375, 2.402 ^b^, 2.495 ^a^
Zn–C_CO_	2.118	2.060	2.088	2.088, 2.100	2.065, 2.079	2.067, 2.090, 2.076, 2.099
Zn–Cp_centroid_	1.960	-	1.959	1.960	-	1.958
Zn–C_Ph_	-	1.973	1.974	-	1.972	1.973
C–O	1.134	1.137	1.135	1.135, 1.138	1.137, 1.139	1.136, 1.137, 1.139
Zn–Zn–Zn	138.5	129.4	133.9	134.0	129.9	130.7, 133.1
C_CO_–Zn–C_CO_	91.9	94.0	92.9	92.5	92.9	92.4, 93.0
Zn–Zn–Cp_centroid_	177.3	-	177.1	176.4	-	176.6
Zn–Zn–C_Ph_	-	176.4	176.8	-	176.8	176.7

^a^ Zn1–Zn2. ^b^ Zn1–Zn4.

**Table 2:** Selected local and integrated topological properties of the electron density at the Zn–Zn BCP for complexes **1**–**6** ^a^.

Complex	Zn–Zn	ρ_BCP_	▽^2^ρ_BCP_	|V_BCP_|/G_BCP_	G_BCP_/ρ_BCP_	H_BCP_/ρ_BCP_	ε_BCP_	δ(Zn,Zn)	WBI	∫_Zn∩Zn_ρ
**1**	2.386	0.058	0.041	1.633	0.483	−0.306	0.013	0.746	0.84	0.678
**2**	2.416	0.057	0.027	1.724	0.432	−0.313	0.032	0.733	0.69	0.661
**3**	2.392 2.413	0.057 0.058	0.041 0.027	1.625 1.737	0.484 0.428	−0.303 −0.315	0.019 0.024	0.729 0.749	0.84 0.70	0.679 0.659
**4**	2.375 2.491	0.059 0.048	0.044 0.023	1.618 1.719	0.492 0.424	−0.304 −0.305	0.017 0.001	0.781 0.750	0.86 0.59	0.682 0.674
**5**	2.403 2.498	0.058 0.047	0.028 0.024	1.720 1.701	0.436 0.429	−0.314 −0.301	0.026 0.004	0.770 0.496	0.72 0.60	0.664 0.667
**6**	2.375 2.402 2.495	0.058 0.059 0.048	0.043 0.029 0.023	1.618 1.718 1.709	0.491 0.436 0.427	−0.304 −0.314 −0.303	0.017 0.027 0.006	0.778 0.774 0.498	0.86 0.72 0.59	0.682 0.666 0.672

^a^ All dimensions are expressed in atomic units (a.u.) except distances, which are in Å. δ(Zn,Zn) means the delocalization index between Zn bonded atoms. ε_BCP_ denotes ellipticity at the BCP. |V_BCP_|/G_BCP_ is an adimensional ratio. G_BCP_/ρ_BCP_ and H_BCP_/ρ_BCP_ are the potential and total energy ratios at the BCP, respectively. WBI is the Wiberd bond index derived from the NBO calculation. ∫_Zn∩Zn_ρ accounts for the integrated electron density over the whole Zn–Zn interatomic surface.

**Table 3:** Selected structural parameters (bond distances, Å, and angles, °) of complexes **7** and **8**.

	7	8
Zn–Zn	2.430	2.445, 2.459, 2.495
Zn–C_CO_	1.973	2.050–2.062
C–O	1.141	1.138–1.140
Zn–Zn–Zn	60.0	104.6, 106.6, 107.2
C_CO_–Zn–C_CO_	105.7	95.8, 95.9, 96.7

**Table 4:** Selected local and integrated topological properties of the electron density at the Zn–Zn BCP for complexes **7**, **8** and [Zn_2_(CO)_4_] ^a^.

Complex	Zn–Zn	ρ_BCP_	▽^2^ρ_BCP_	|V_BCP_|/G_BCP_	G_BCP_/ρ_BCP_	H_BCP_/ρ_BCP_	ε_BCP_	δ(Zn,Zn)	WBI	∫_Zn∩Zn_ρ
**7**	2.430	0.052	0.045	1.567	0.500	−0.283	0.641	0.552	0.59	0.556
**8**	2.445 2.459 2.495	0.051 0.050 0.047	0.032 0.031 0.025	1.659 1.658 1.687	0.451 0.450 0.431	−0.297 −0.296 −0.296	0.036 0.033 0.051	0.593 0.552 0.497	0.65 0.62 0.55	0.634 0.633 0.634
[Zn_2_(CO)_4_]	2.278	0.067	0.084	1.481	0.609	−0.316	0.050	0.996	1.09	0.784

^a^ For units and definitions see the footnote of Table 2.

**Table 5:** Source Function contributions (%) to the electron density at Zn–Zn BCPs for **1**–**8** ^a^.

Complex	%SF_ba_	%SF_Zn_	%SF_O_
**1**	42.19–36.39 Zn_Cp_-Zn_CO_	1.73	6.45
**2**	43.89–36.40 Zn_Ph_-Zn_CO_	2.31	6.56
**3**	41.87–35.61 Zn_Cp_-Zn_CO_ 44.04–37.22 Zn_Ph_-Zn_CO_	2.33 1.68	6.77 6.28
**4**	42.27–37.16 Zn_Cp_-Zn_CO_ 33.29–33.29 Zn_Ph_-Zn_CO_	0.73/0.16 2.06	6.13 7.77
**5**	44.06–37.88 Zn_Ph_-Zn_CO_ 32.53–32.53 Zn_Ph_-Zn_CO_	0.62/0.35 2.77	6.06 8.09
**6**	42.17–37.19 Zn_Cp_-Zn_CO_ 44.07–37.94 Zn_Ph_-Zn_CO_ 32.70–33.06 Zn_Ph_-Zn_CO_	0.72/0.36 0.69/0.14 2.07/2.074	6.14 6.04 7.93
**7**	31.10–31.10 * 22.40	7.86	6.53 4.03
**8**	34.09–34.09 (aver) * −9.78 (aver)	[−0.15–1.08]	7.02 (aver) 2.01 (aver)

^a^ SF_ba_ means the SF contribution from bonded atoms. SF_Zn_ implies SF contribution from the metal atoms not involved in the BCP taken as reference point. SF_O_ means the SF contribution from the O atoms of the Zn(CO)_2_ unit participating in the BCP taken as reference. (aver) means the average value of the different Zn–Zn bonds present in the complex **8**. Individual values are rather close to the average one. * SF_ba_ value is an average value among all the Zn atoms and takes the RCP as a reference. Individual values are rather close to the average one.

**Table 6:** Average electron population in polysynaptic metal basins ^a^.

Complex	V(Zn_Cp_-Zn_CO_)	V(Zn_Ph_-Zn_CO_)	V(Zn_CO_-Zn_CO_)
**1**	2.38		
**2**		2.23	
**3**	2.33	2.28	
**4**	2.45		1.61
**5**		2.35	1.50
**6**	2.45	2.36	1.55
**7**			1.23
**8**			1.56 1.71 (×2) 1.84 (×2)
[Zn_2_(CO)_4_]			3.05

^a^ For the sake of comparison, the average electron population for the V(Zn,Zn) basin of [Zn_2_(CO)_4_] has also been included.

## Data Availability

Data are available from the authors upon request.

## Supplementary Materials

The following supporting information can be downloaded at: https://www.mdpi.com/article/10.3390/ijms232314858/s1.
