# Supplementary material for: Theoretical Analysis of Polynuclear Zinc Complexes Isolobally Related to Hydrocarbons
Source: Int J Mol Sci. 2022 Nov 28;23(23):14858. doi: 10.3390/ijms232314858 (PMC9736195; doi:10.3390/ijms232314858)
Supplement: Supplementary file 1 [file ijms-23-14858-s001.zip › ijms-2035002-supplementary.pdf]

International Journal of Molecular Sciences (ISSN 1422-0067)

Special Issue "Multi-Metallic Systems: From Strong Cooperative Bonds to Weak M-M Interactions"

Supplementary Material

Theoretical Analysis of Polynuclear Zinc Complexes Isolobally Related to Hydrocarbons

Regla Ayala and Agustín Galindo

Figure S1. Histogram of the Zn-Zn distances found in structurally characterized dizinc complexes.

Figure S2. MOs of complexes **1-3** involved in the Zn-Zn interactions.

Figure S3. MOs of complexes **4-6** involved in the Zn-Zn interactions.

Figure S4. Topological graphs showing bond paths (BPs) and critical points (CPs) for complexes **1-8**.

Figure S5. MOs of complexes **7** and **8** involved in the Zn-Zn interactions.

Figure S6. Two-dimensional (2D) ELF cross sections through the complexes **1-8**.

Figure S7. Three-dimensional (3D) representation at the ELF isosurface value of 0.47 for complexes **1-8**.

Table S1. Selected IR bands of complexes **1-8** (cm^{-1}, not corrected).

Table S2. Comparison between MP2 and TPSSh results.

Table S3. Coordinates of the optimized compounds at TPSSh/Def2TZVPP level.

Figure S1. Histogram of the Zn-Zn distances found in structurally characterized dizinc complexes.

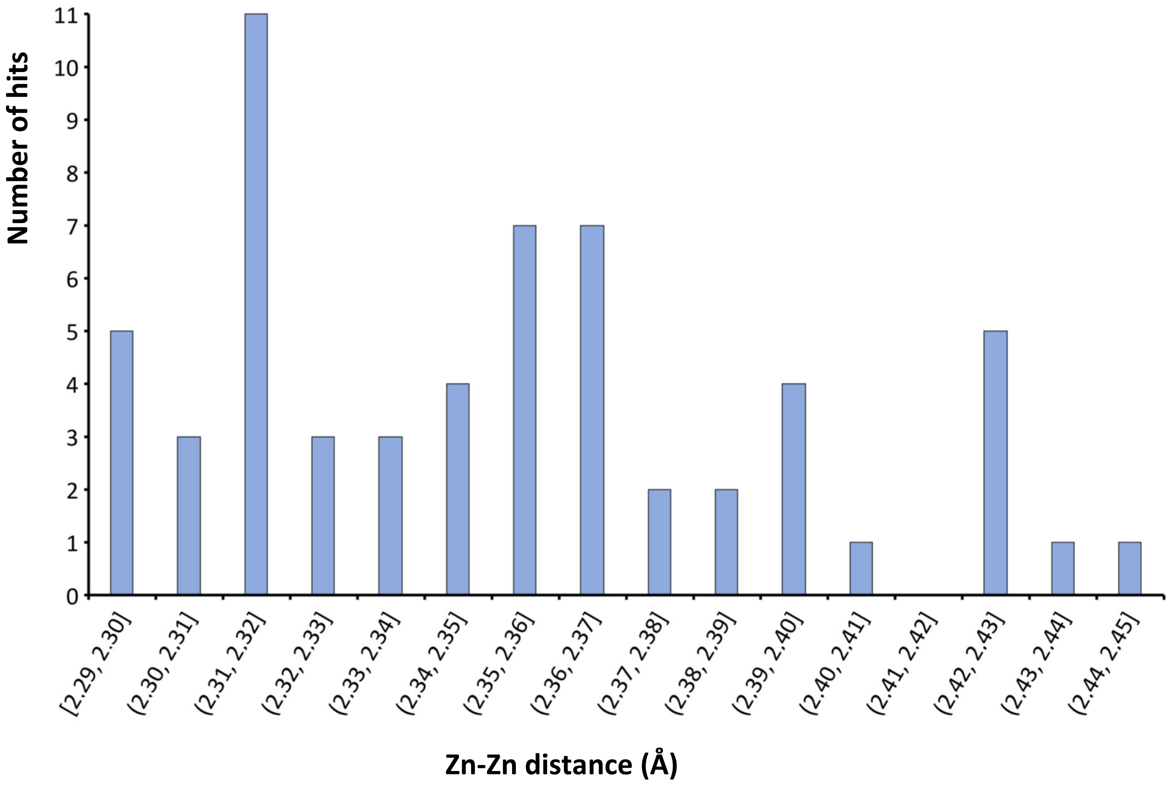

Figure S2. MOs of complexes **1-3** involved in the Zn-Zn interactions.

Complex **1**

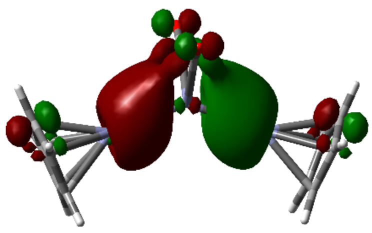

HOMO (MO 94, -0.20224)

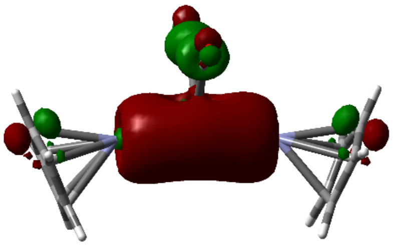

HOMO-5 (MO 89, -0.24118)

MOs composition (Zn orbitals):
94 is Zn1-p=0.3030 Zn2-s=0.1864 Zn3-s=0.1864
89 is Zn1-s=0.2286 Zn3-s=0.1345 Zn2-s=0.1345 Zn1-p=0.1232 Zn3-p=0.0695 Zn2-p=0.0695

Complex **2**

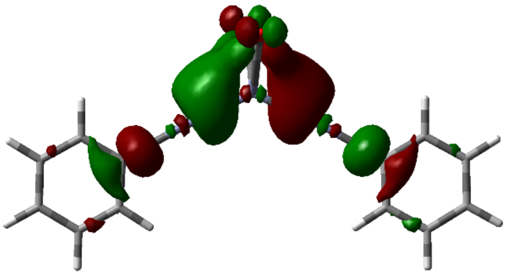

HOMO (MO 100, -0.19206)

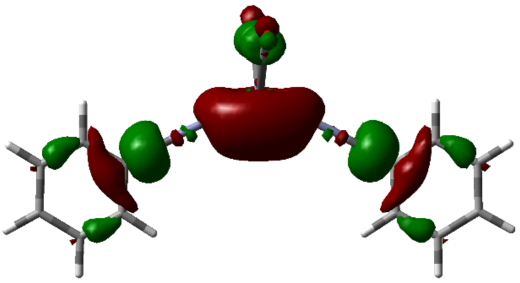

HOMO-1 (MO 99, -0.22443)

MOs composition (Zn orbitals):
100 is Zn1-p=0.2602 Zn2-p=0.1051 Zn3-p=0.1050 Zn3-s=0.0397 Zn2-s=0.0397
99 is Zn1-s=0.1740 Zn3-p=0.1243 Zn2-p=0.1242 Zn1-p=0.0884

Complex **3**

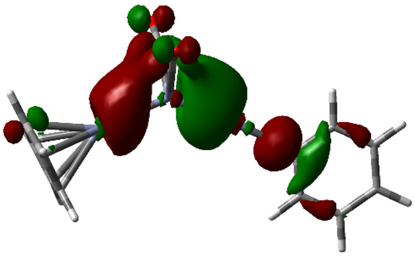

HOMO (MO 97, -0.19559)

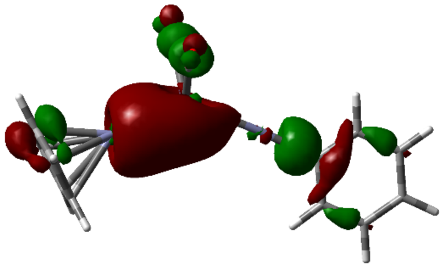

HOMO-3 (MO 94, -0.23367)

MOs composition (Zn orbitals):
97 is Zn1-p=0.2746 Zn2-p=0.1522 Zn3-s=0.1090 Zn2-s=0.0506
94 is Zn1-s=0.1896 Zn3-s=0.1844 Zn1-p=0.1133 Zn2-p=0.0869 Zn3-p=0.0756

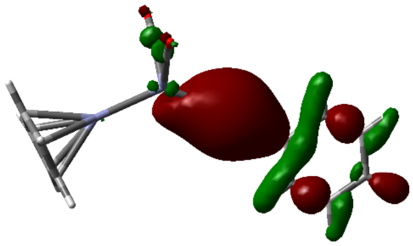

HOMO-6 (MO 91, -0.28146)

MOs composition (Zn orbitals):
91 is Zn2-s=0.5367 Zn1-p=0.0255 Zn1-s=0.0253 Zn2-d=0.0205

Figure S3. MOs of complexes **4-6** involved in the Zn-Zn interactions.

Complex **4**

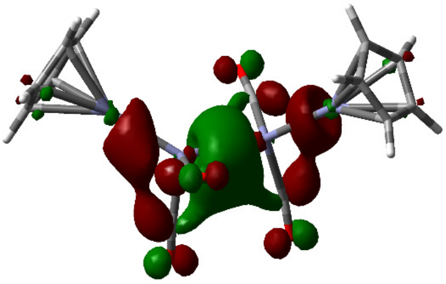

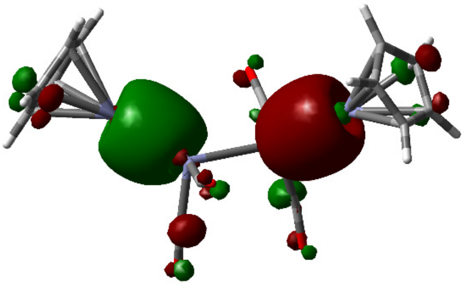

HOMO (MO 123, -0.18301) HOMO-5 (MO 118, -0.22643)

MOs composition (Zn orbitals):
123 is Zn1-p=0.1590 Zn2-p=0.1590 Zn3-s=0.1117 Zn4-s=0.1117 Zn2-s=0.0199 Zn1-s=0.0199 Zn4-p=0.0143 Zn3-p=0.0143
118 is Zn3-s=0.1637 Zn4-s=0.1637 Zn1-p=0.1043 Zn2-p=0.1042 Zn1-s=0.0782 Zn2-s=0.0782 Zn3-p=0.0513 Zn4-p=0.0513

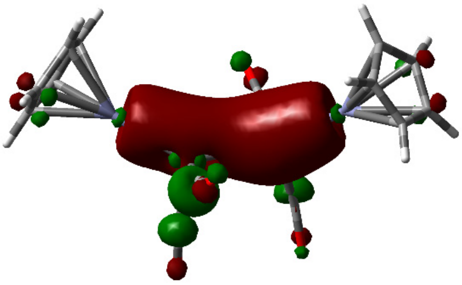

HOMO-6 (MO 117, -0.24046)

MOs composition (Zn orbitals):
117 is Zn2-s=0.1802 Zn1-s=0.1802 Zn2-p=0.0840 Zn1-p=0.0840 Zn3-s=0.0707 Zn4-s=0.0707 Zn3-p=0.0289 Zn4-p=0.0289 Zn2-d=0.0147 Zn1-d=0.0147

Complex **5**

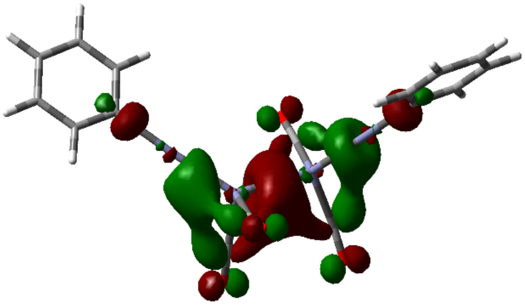

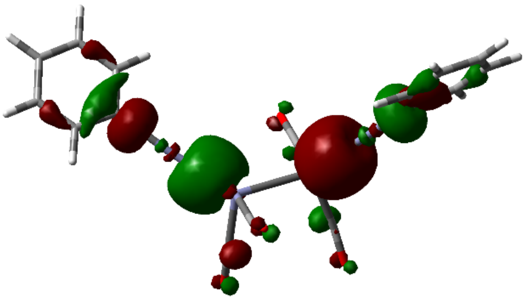

HOMO (MO 129, -0.19213) HOMO-1 (MO 128, -0.22831)

MOs composition (Zn orbitals):
129 is Zn2-p=0.1589 Zn1-p=0.1589 Zn3-p=0.0553 Zn4-p=0.0553 Zn4-s=0.0456 Zn3-s=0.0456
128 is Zn4-p=0.1200 Zn3-p=0.1200 Zn1-p=0.0752 Zn2-p=0.0752 Zn2-s=0.0611 Zn1-s=0.0611 Zn3-s=0.0203 Zn4-s=0.0203

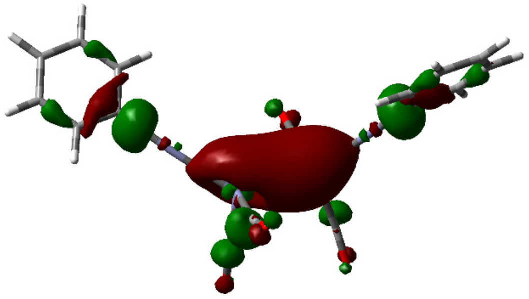

HOMO-2 (MO 127, -0.25388)

MOs composition (Zn orbitals):
127 is Zn1-s=0.1575 Zn2-s=0.1575 Zn2-p=0.0767 Zn1-p=0.0767 Zn3-p=0.0596 Zn4-p=0.0596 Zn4-s=-0.0109 Zn3-s=-0.0109 Zn2-d=0.0103 Zn1-d=0.0103

Complex **6**

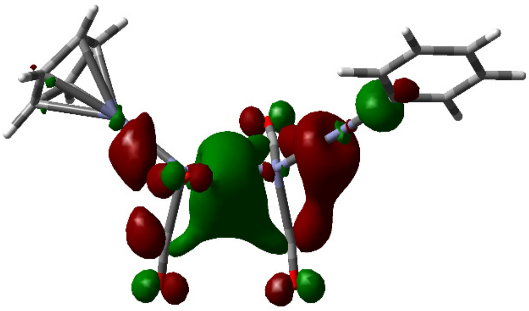

HOMO (MO 126, -0.18226)

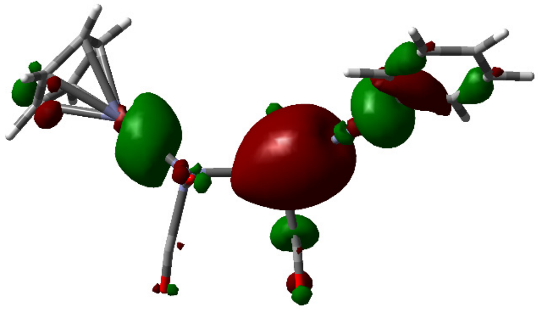

HOMO-1 (MO 125, -0.21611)

MOs composition (Zn orbitals):
126 is Zn1-p=0.1821 Zn2-p=0.1369 Zn3-s=0.0869 Zn4-p=0.0681 Zn4-s=0.0564 C5-p=0.0513 Zn2-s=0.0176 Zn3-p=0.0111
125 is Zn4-p=0.1515 Zn1-s=0.1445 Zn3-s=0.0982 Zn1-p=0.0912 Zn2-p=0.0747 Zn3-p=0.0266

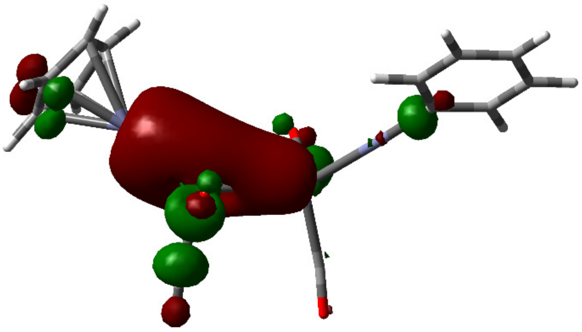

HOMO-6 (MO 120, -0.23865)

MOs composition (Zn orbitals):
120 is Zn2-s=0.2520 Zn3-s=0.1464 Zn2-p=0.1247 Zn1-s=0.0748 Zn3-p=0.0559 Zn1-p=0.0454 Zn4-p=0.0149 Zn2-d=0.0146

Figure S4. Topological graphs showing bond paths (BPs) and critical points (CPs) for complexes **1-8**.

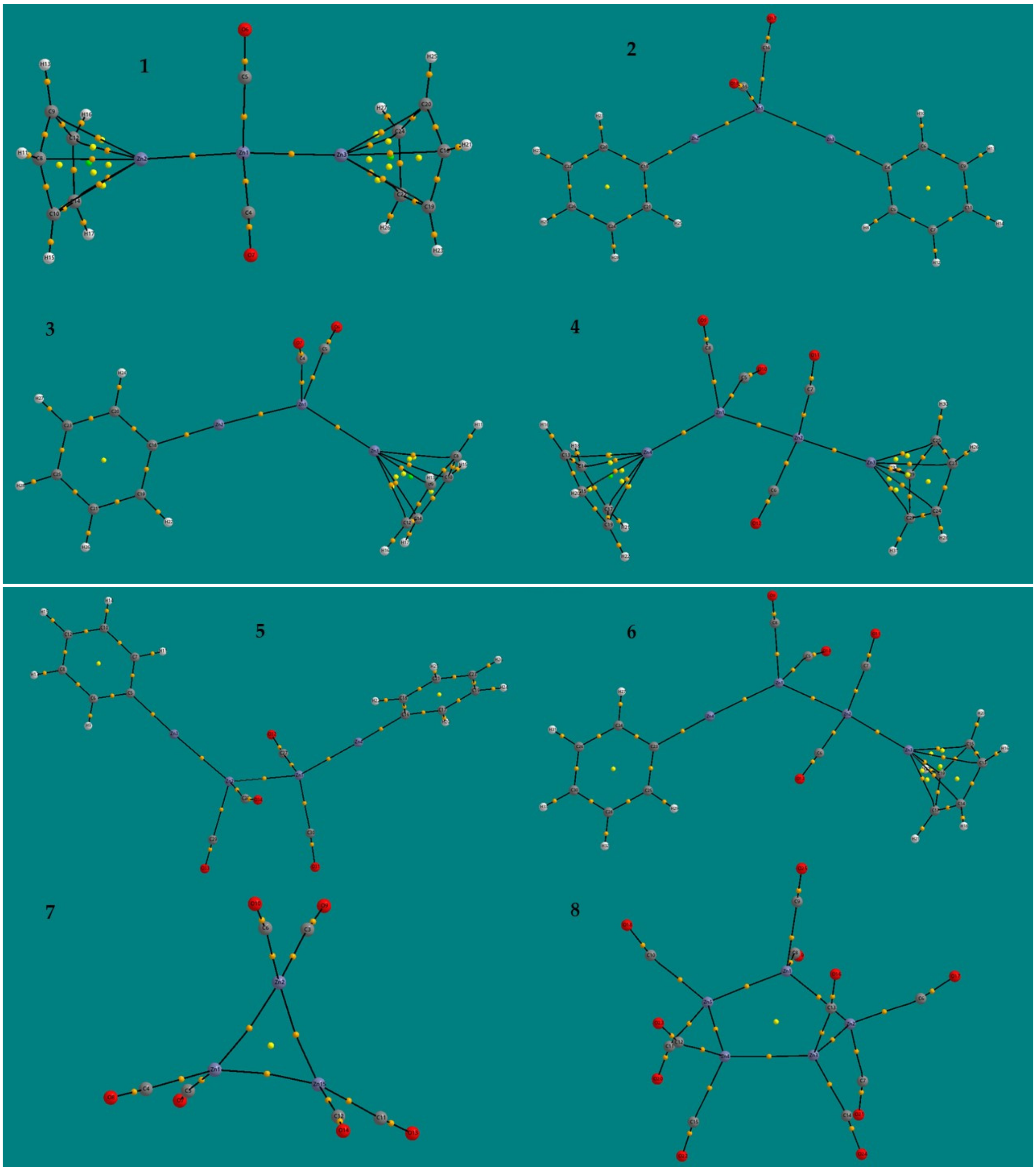

Color criteria: BPs (black); BCPs (orange); RCPs (yellow); CCPs (green).

Figure S5. MOs of complexes **7** and **8** involved in the Zn-Zn interactions.

Complex **7**

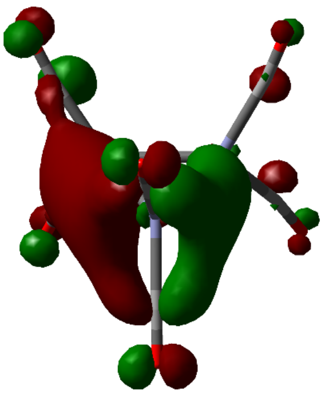

HOMO (MO 87, -0.16329)

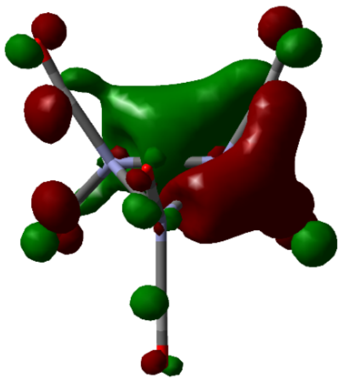

HOMO-1 (MO 86, -0.16342)

MOs composition (Zn orbitals):
87 is Zn2-p=0.2684 Zn15-p=0.1484 Zn1-s=0.0397 Zn1-p=0.0316 Zn15-s=0.0214
86 is Zn1-p=0.2676 Zn15-p=0.1507 Zn2-s=0.0406 Zn2-p=0.0307 Zn15-s=0.0216

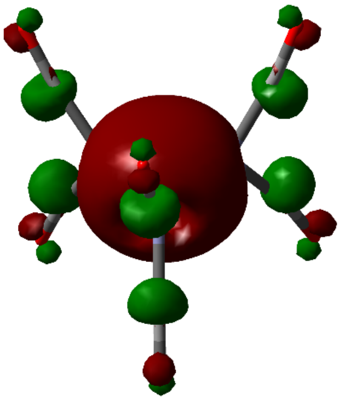

HOMO-2 (MO 85, -0.28021)

MOs composition (Zn orbitals):
85 is Zn1-p=0.1287 Zn15-p=0.1284 Zn2-p=0.1280 Zn1-s=0.1156 Zn15-s=0.1153 Zn2-s=0.1141

Complex **8**

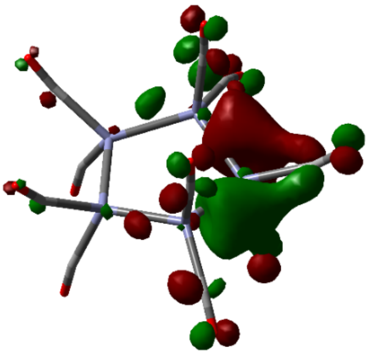

HOMO (145, -0.16525)

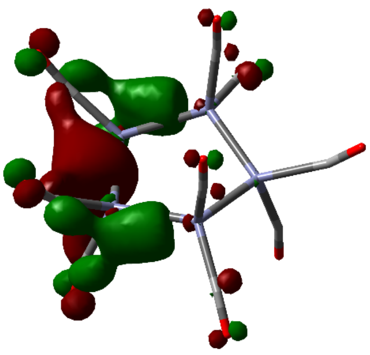

HOMO-1 (144, -0.16699)

MOs composition (Zn orbitals):
145 is Zn2-p=0.1867 Zn1-p=0.1191 Zn3-p=0.1182 Zn5-p=0.0203 Zn4-p=0.0196 Zn4-s=0.0145 Zn5-s=0.0144
144 is Zn4-p=0.1755 Zn5-p=0.1747 Zn3-p=0.0562 Zn1-p=0.0552 Zn1-s=0.0103 Zn3-s=0.0102

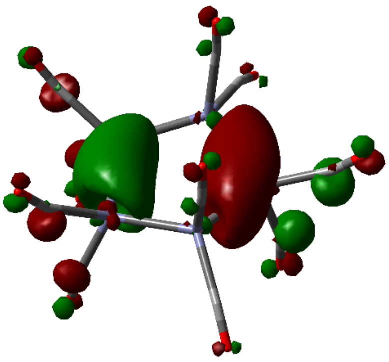

HOMO-2 (143, -0.19853)

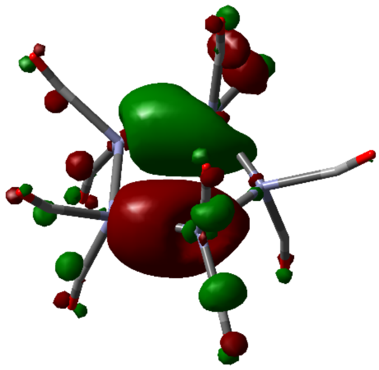

HOMO-3 (142, -0.20130)

MOs composition (Zn orbitals):
143 is Zn2-s=0.1509 Zn4-s=0.0819 Zn5-s=0.0818 Zn1-p=0.0794 Zn3-p=0.0793 Zn2-p=0.0568 Zn4-p=0.0436 Zn5-p=0.0435 Zn2-d=0.0132 Zn4-d=0.0107 Zn5-d=0.0107
142 is Zn3-s=0.1271 Zn1-s=0.1270 Zn4-p=0.0693 Zn5-p=0.0692 Zn2-p=0.0637 Zn1-p=0.0567 Zn3-p=0.0566 Zn5-s=0.0500 Zn4-s=0.0499 Zn1-d=0.0123 Zn3-d=0.0123

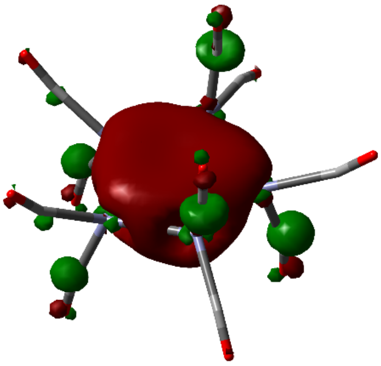

HOMO-4 (141, -0.27426)

141 is Zn3-s=0.0847 Zn1-s=0.0847 Zn4-s=0.0812 Zn5-s=0.0811 Zn2-s=0.0728 Zn4-p=0.0708 Zn5-p=0.0708 Zn3-p=0.0700 Zn1-p=0.0700 Zn2-p=0.0641

Figure S6. Two-dimensional (2D) ELF cross sections through the complexes **1-8**.

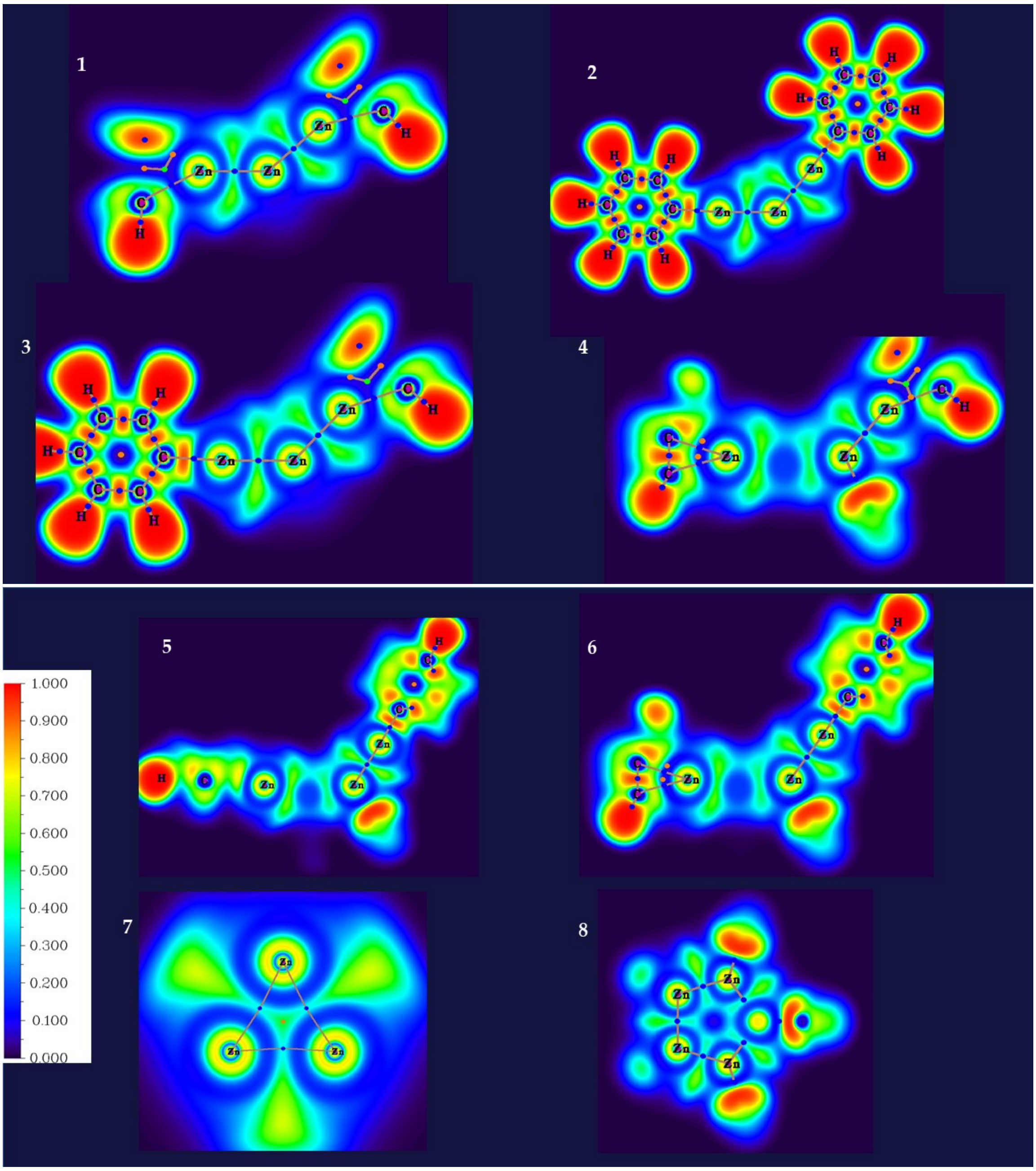

For the sake of completeness, BPs and CPs in the cross sections here studied also appear in the figure.

Figure S7. Three-dimensional (3D) representation at the ELF isosurface value of 0.47 for complexes **1-8**.

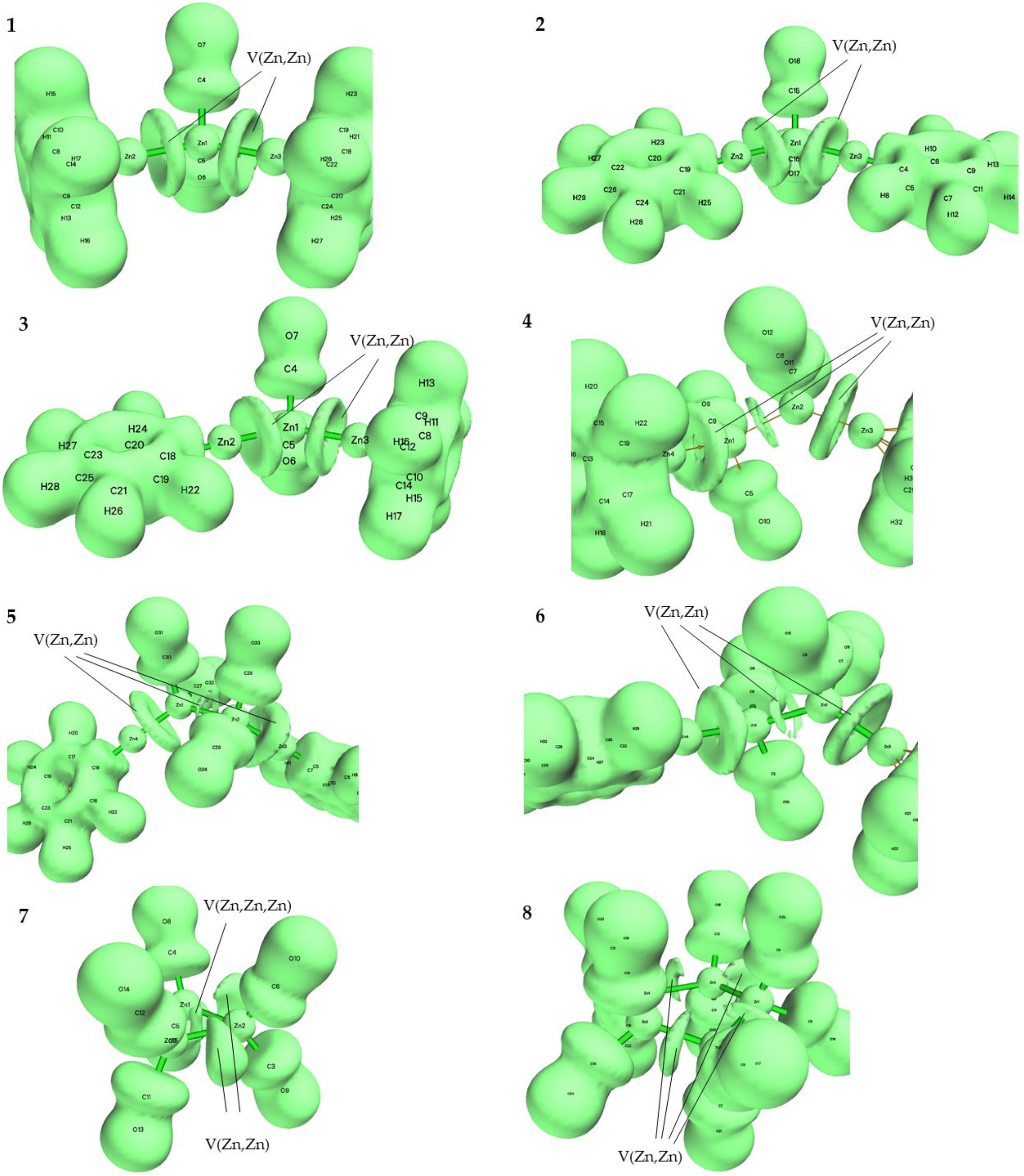

The polysynaptic basins involving metal domains have been labeled in the figure.

Table S1. Selected IR bands of complexes **1-8** (cm^{-1}, not corrected).

**Complex**	**1**	**2**	**3**	**4**	**5**	**6**	**7**	**8**
ν(CO)	2124, 2111	2106, 2087	2114, 2099	2122, 2098, 2090, 2065	2114, 2085, 2078, 2057	2118, 2093, 2083, 2059	2058	2083, 2077
ν(ZnZn)	334, 319	312, 302	326, 305	332, 328, 213	311, 310, 207	330, 311, 211	355, 206, 159	275, 207, 204, 172, 171, 150

Free CO: ν(CO) = 2143 cm^{-1}.

Table S2. Comparison between TPSSh and MP2 results.

Complexes	**1**	**1**	**2**	**2**	**3**	**3**	**4**	**4**	**5**	**5**	**7**	**7**
	**TPSSh**	**MP2**	**TPSSh**	**MP2**	**TPSSh**	**MP2**	**TPSSh**	**MP2**	**TPSSh**	**MP2**	**TPSSh**	**MP2**
Zn-Zn	2.386	2.364	2.416	2.392	2.392, 2.413	2.370, 2.389	2.375, 2.491	2.350, 2.451	2.403, 2.498	2.376, 2.463	2.430	2.392
Zn-C_{CO}	2.118	2.152	2.060	2.064	2.088	2.105	2.088, 2.100	2.075, 2.096	2.065, 2.079	2.049, 2.073	1.973	1.936
Zn-$Cp_{centroid}$	1.960	1.931	-	-	1.959	1.929	1.960	1.932	-	-	-	-
Zn-C_{Ph}	-	-	1.973	1.946	1.974	1.947	-	-	1.972	1.946	-	-
C-O	1.134	1.138	1.137	1.140	1.135	1.139	1.135, 1.138	1.140, 1.144	1.137, 1.139	1.141, 1.145	1.141	1.148
Zn-Zn-Zn	138.5	141.1	129.4	129.4	133.9	135.3	134.0	135.1	129.9	130.5	60.0	60.0
C_{CO}-Zn-C_{CO}	91.9	88.99	94.0	92.60	92.9	90.7	92.5	91.9	92.9	92.1	105.7	107.8
Zn-Zn-$Cp_{centroid}$	177.3	176.7	-	-	177.1	176.5	176.4	173.4	-	-	-	-
Zn-Zn-C_{Ph}	-	-	176.4	176.9	176.8	177.3	-	-	176.8	176.0	-	-

Bond distances in Å and angles in °.

Table S3. Coordinates of the optimized compounds at TPSSh/Def2TZVPP level.

[(ZnCp)$_2${μ-Zn(CO)$_2$}], **1**.

Zn	0.00001000	0.61028300	-0.00015600
Zn	-2.23157900	-0.23401600	-0.00068900
Zn	2.23159100	-0.23402100	-0.00063600
C	-0.00008200	2.08056900	1.52430900
C	0.00000400	2.08394600	-1.52128700
O	0.00000300	2.60360800	-2.52945300
O	-0.00016500	2.59811900	2.53356200
C	-4.51596300	0.09524400	-0.01840900
C	-4.17327700	-0.68530000	-1.15506800
C	-4.18681900	-0.65315000	1.14353100
H	-4.95710200	1.08041200	-0.03479300
C	-3.63440600	-1.91801700	-0.69545500
H	-4.31066700	-0.39870000	-2.18677600
C	-3.64272800	-1.89812400	0.72494300
H	-4.33619500	-0.33780900	2.16514000
H	-3.28833700	-2.73016900	-1.31666200
H	-3.30413300	-2.69253100	1.37265200
C	4.51601300	0.09537500	-0.01103200
C	4.18407700	-0.65953400	1.14589600
C	4.17609200	-0.67877400	-1.15287200
H	4.95716300	1.08062600	-0.02081400
C	3.64103700	-1.90214700	0.71901500
H	4.33098600	-0.34993200	2.16961700
C	3.63612900	-1.91405600	-0.70148900
H	4.31595700	-0.38638200	-2.18262100
H	3.30089500	-2.70018000	1.36143200
H	3.29159100	-2.72271400	-1.32808200

[(ZnPh)$_2${μ-Zn(CO)$_2$}], **2**.

Zn	0.00003600	1.17748800	-0.00010400
Zn	-2.18474900	0.14489600	-0.00192600
Zn	2.18481000	0.14494000	0.00159800
C	3.91237700	-0.80782100	0.00435500
C	3.97725200	-2.20841400	0.08244100
C	5.13203600	-0.11608600	-0.07109800
C	5.19458600	-2.88629200	0.08534900
H	3.06403400	-2.79437900	0.14282000
C	6.35465900	-0.78437600	-0.06901800
H	5.14089300	0.96888600	-0.13329100
C	6.38765100	-2.17358000	0.00943500
H	5.21264000	-3.96923500	0.14673700
H	7.28065300	-0.22233400	-0.12846200
H	7.33691600	-2.69735300	0.01139600
C	-0.00049200	2.58261600	1.50626200
C	0.00023100	2.58297900	-1.50614700
O	0.00018500	3.13391800	-2.50045200
O	-0.00069600	3.13322000	2.50075400
C	-3.91233000	-0.80785100	-0.00443700
C	-5.13196600	-0.11619200	0.07203100
C	-3.97720600	-2.20840500	-0.08322500
C	-6.35457200	-0.78451700	0.07026300
H	-5.14082100	0.96874800	0.13478500
C	-5.19452100	-2.88631700	-0.08583500
H	-3.06400200	-2.79431200	-0.14440500
C	-6.38756500	-2.17367900	-0.00889600
H	-7.28055000	-0.22253300	0.13050500
H	-5.21257900	-3.96922800	-0.14778500
H	-7.33681600	-2.69748000	-0.01061400

[(ZnPh){μ-Zn(CO)$_2$}(ZnCp)], **3**.

Zn	0.51917900	0.81773600	-0.00022500
Zn	-1.81576300	0.20942500	0.00015900
Zn	2.55949800	-0.43033700	0.00015700
C	0.83695300	2.22232600	1.51183900
C	0.83625900	2.22054100	-1.51411600
O	0.96670800	2.73906400	-2.51580700
O	0.96813300	2.74227900	2.51269000
C	4.86275800	-0.54124300	-0.00564500
C	4.39098700	-1.22312000	1.14818800
C	4.38461000	-1.23329500	-1.15078300
H	5.48280600	0.34241800	-0.01127400
C	3.62326200	-2.33909200	0.71602400
H	4.59206700	-0.95046400	2.17318400
C	3.61934100	-2.34538900	-0.70451800
H	4.58002200	-0.96973200	-2.17924700
H	3.13729300	-3.06056300	1.35521400
H	3.12985300	-3.07246500	-1.33460400
C	-3.69525500	-0.39234400	0.00029400
C	-4.03105900	-1.75603900	-0.00125900
C	-4.75837600	0.52521400	0.00187200
C	-5.35695400	-2.18445500	-0.00124700
H	-3.24860100	-2.51019200	-0.00251800
C	-6.08769700	0.10756200	0.00190200
H	-4.55691100	1.59326800	0.00311200
C	-6.38952900	-1.25114200	0.00033700
H	-5.58466200	-3.24516500	-0.00247400
H	-6.88729500	0.84078900	0.00314300
H	-7.42251700	-1.58047500	0.00034900

[(ZnCp)$_2${μ-Zn$_2$(CO)$_4$}], **4**.

Zn	-1.12808800	0.66410700	-0.52795700
Zn	1.12809300	0.66411600	0.52792100
Zn	3.15085400	-0.50319000	0.09870400
Zn	-3.15084300	-0.50320600	-0.09872200
C	-0.51208600	0.34394900	-2.51036800
C	0.51204200	0.34390300	2.51030600
C	1.46946900	2.69557800	0.86732700
C	-1.46948300	2.69558400	-0.86728200
O	-1.59844000	3.81622700	-0.71958400
O	-0.05130300	-0.09748900	-3.44950900
O	1.59842300	3.81623300	0.71972200
O	0.05123800	-0.09757500	3.44941800
C	-5.44824700	-0.56486400	0.02403800
C	-5.02391500	-1.62772300	-0.81784400
C	-4.93484800	-0.80560500	1.32761900
H	-6.06257400	0.27211700	-0.27146900
C	-4.24804100	-2.52526700	-0.03450700
H	-5.25948300	-1.74045800	-1.86524900
C	-4.19480300	-2.01766600	1.29136900
H	-5.09137100	-0.18345700	2.19582800
H	-3.79121400	-3.43917100	-0.38291200
H	-3.68709700	-2.47629200	2.12634800
C	5.44823400	-0.56467200	-0.02448900
C	5.02418300	-1.62726200	0.81787400
C	4.93460100	-0.80595600	-1.32787800
H	6.06253100	0.27248500	0.27058100
C	4.24822900	-2.52516900	0.03503400
H	5.25997400	-1.73958100	1.86527300
C	4.19468100	-2.01807400	-1.29102300
H	5.09088800	-0.18411800	-2.19635100
H	3.79155900	-3.43898600	0.38387500
H	3.68685500	-2.47706700	-2.12572900

[(ZnPh)$_2${μ-Zn$_2$(CO)$_4$}], **5**.

Zn	-0.60502000	-1.09261200	1.17116600
Zn	0.60502000	1.09261200	1.17116600
Zn	0.29544600	3.02564000	-0.22314600
Zn	-0.29544600	-3.02564000	-0.22314600
C	-0.04733700	4.56182200	-1.41119000
C	0.77382800	5.70087300	-1.39304500
C	-1.12288700	4.56610300	-2.31424100
C	0.53701800	6.78869000	-2.23088000
H	1.62071000	5.75176000	-0.71384600
C	-1.36857900	5.64870600	-3.15629900
H	-1.79091900	3.71080700	-2.37110100
C	-0.53701800	6.76404500	-3.11576200
H	1.18944000	7.65464600	-2.19378700
H	-2.20757800	5.62233700	-3.84351900
H	-0.72470500	7.60839100	-3.76947000
C	0.04733700	-4.56182200	-1.41119000
C	-0.77382800	-5.70087300	-1.39304500
C	1.12288700	-4.56610300	-2.31424100
C	-0.53701800	-6.78869000	-2.23088000
H	-1.62071000	-5.75176000	-0.71384600
C	1.36857900	-5.64870600	-3.15629900
H	1.79091900	-3.71080700	-2.37110100
C	0.53701800	-6.76404500	-3.11576200
H	-1.18944000	-7.65464600	-2.19378700
H	2.20757800	-5.62233700	-3.84351900
H	0.72470500	-7.60839100	-3.76947000
C	-2.54708600	-0.36513300	1.09184300
C	2.54708600	0.36513300	1.09184300
C	0.74117900	1.54098700	3.18648300
C	-0.74117900	-1.54098700	3.18648300
O	-0.51144100	-1.77737100	4.27637000
O	-3.51869000	0.14054800	0.78733800
O	0.51144100	1.77737100	4.27637000
O	3.51869000	-0.14054800	0.78733800

[(ZnPh){μ-Zn(CO)$_2$}$_2$(ZnCp)], **6**.

Zn	0.65434400	1.14713700	0.44207100
Zn	-1.51719700	0.54726800	-0.62965200
Zn	-3.28560200	-0.91065900	-0.00646900
Zn	2.82016000	0.12265700	0.27087800
C	-0.04955700	1.22322100	2.39342900
C	-0.82696700	0.04390000	-2.54791300
C	-2.24286900	2.39672100	-1.27849100
C	0.67677500	3.20413300	0.25505500
O	0.66720300	4.27040800	-0.14485500
O	-0.50172900	0.97662200	3.40674700
O	-2.59529000	3.47793100	-1.31020300
O	-0.29198300	-0.45149600	-3.41854800
C	-5.52963700	-1.39569200	0.12956200
C	-4.88467900	-2.47711600	-0.52865100
C	-5.01781000	-1.31950200	1.45370700
H	-6.28385000	-0.75205800	-0.29724000
C	-3.97402400	-3.06924000	0.38871700
H	-5.06312500	-2.79983100	-1.54318200
C	-4.05791100	-2.35458400	1.61386400
H	-5.31577000	-0.60810500	2.20900000
H	-3.34009200	-3.92078400	0.19298200
H	-3.49617800	-2.56564100	2.51118200
C	4.55733400	-0.78500200	0.04827000
C	5.69878700	-0.36907000	0.75269800
C	4.70907100	-1.87656700	-0.82228100
C	6.92820800	-1.00649100	0.59888500
H	5.63920400	0.47055900	1.44016700
C	5.93384000	-2.52112000	-0.98354400
H	3.85911900	-2.24125900	-1.39297200
C	7.04774500	-2.08596100	-0.27152000

H	7.79212100	-0.66179300	1.15726000
H	6.01939700	-3.36190900	-1.66375700
H	8.00256200	-2.58476800	-0.39409900

[$Zn_3(CO)_6$], **7**.

Zn	-1.30101200	-0.52736600	0.00773700
Zn	0.19414800	1.38770900	0.00268500
Zn	1.10395200	-0.86560000	-0.01198500
C	0.34880200	2.57663100	-1.56420600
C	-2.39446600	-0.97282800	1.58886600
C	-2.42067900	-0.96303100	-1.55752500
C	0.37829400	2.55538400	1.58286800
O	-2.92810600	-1.15811700	-2.56091300
O	-2.88466400	-1.17388500	2.59961600
O	0.41513200	3.11443600	-2.56857400
O	0.46489700	3.07825000	2.59356900
C	2.03229400	-1.58734600	-1.59684800
C	2.05996800	-1.60279400	1.54872100
O	2.44705900	-1.91004000	-2.60985100
O	2.49344500	-1.93544300	2.55060800

[$Zn_5(CO)_{10}$], **8**.

Zn	0.68307000	1.94643500	0.21482500
Zn	2.08689100	0.00208000	-0.32966000
Zn	0.68682800	-1.94506300	0.21491600
Zn	-1.64403100	-1.24913000	-0.03171300
Zn	-1.64639300	1.24631800	-0.03299900
C	4.04955300	0.00390300	0.27717500
C	2.46076700	0.00207300	-2.34526400
C	1.27163700	3.75327500	-0.58494400
C	1.05127400	2.45145700	2.17466300
C	-3.10042600	2.00674200	1.20781300
C	-2.59265100	1.74057900	-1.78873000
C	-3.09539000	-2.01037500	1.21167100
C	1.05758300	-2.45107800	2.17400900
C	1.27872900	-3.74993800	-0.58682500
C	-2.59114100	-1.74752800	-1.78581100
O	1.17220500	-2.43898100	3.30686000
O	4.98963700	0.00479600	0.92083900
O	-3.71879400	2.26710500	2.12861000
O	1.53569900	4.57942600	-1.32298700
O	-2.90470900	1.77730800	-2.88462900
O	2.36699000	0.00162000	-3.48035900
O	-2.90405600	-1.78636600	-2.88139400
O	-3.71253800	-2.27050100	2.13335200
O	1.54425900	-4.57475100	-1.32584300
O	1.16498300	2.43861400	3.30759800
